# Simulation training in vitreoretinal surgery: a systematic review

**DOI:** 10.1186/s12886-019-1098-x

**Published:** 2019-04-11

**Authors:** Rasmus Christian Rasmussen, Jakob Grauslund, Anna Stage Vergmann

**Affiliations:** 1 Department of Ophthalmology, Odense University Hospital, J.B. Winsløws Vej 4, DK-5000 Odense C, Denmark; 2 Department of Clinical Research, University of Southern Denmark, Winløwsparken 19, DK-5000 Odense C, Denmark

**Keywords:** EYESI, Simulation-based training, Surgical simulator, Vitreoretinal surgery

## Abstract

**Background:**

The purpose of this study was to perform a systematic review of the current literature on simulator-based training in vitreoretinal surgery (VRS). We examined the results regarding simulated VRS and provided an overview of how the current results may be employed in VRS training. Lastly, we evaluated the quality of these results.

**Methods:**

The databases of Pubmed, Embase and Cochrane Library were searched for articles in English involving simulated VRS training. A qualitative analysis was performed, since the studies which met our inclusion criteria did not allow for a quantitative meta-analysis.

**Results:**

We identified 203 articles of which seven met the inclusion criteria. Of these, six studies investigated simulation with EyeSi® Surgical (VRMagic, Mannheim, Germany). Six studies reported positive performance curves. Four studies showed construct validity. One study attempted to show skill transfer from simulator to vitrectomies performed on cadavers. Methodological quality of the included studies was moderate but lacking in instrument validation.

**Conclusion:**

Simulator-based training in VRS can assess and possibly assist acquisition of a variety of VRS skills. Further research is needed to support transfer from simulator to operating room. Future studies should strive to follow established validation frameworks and streamline study designs.

**Electronic supplementary material:**

The online version of this article (10.1186/s12886-019-1098-x) contains supplementary material, which is available to authorized users.

## Background

When it comes to simulation training, aviation stands out as a profession where it has been an immense success. Over the last many decades, simulation training has made it possible for pilots to repeatedly encounter rare situations in a safe environment, thus dramatically reducing flight accidents [1, 2]. Surgical simulators offer the same possibility of practicing basic and complex procedures without endangering patients [3–7]. Training simulators have emerged in numerous surgical fields, including laparoscopic, [8] spinal, [9] cardiac, [10] orthopedic, [3] and ophthalmic simulators [11, 12].

Traditionally resident surgeons have been taught with the apprentice model following the paradigm of “see one, do one, teach one” [13]. Khalifa et al. [14] has stated that the apprentice model is limited in the need of real patients and available time to acquire an increasing number of complex surgical skills. Procedures performed by trainees has increased complications [8, 15]. Furthermore, teaching in the operating room is associated with an increased financial cost [16]. All of which might be avoided by the use of simulators.

Simulator training gives an opportunity to acquire the delicate and complex skill required to perform vitreoretinal surgery (VRS), without compromising patient safety. Nevertheless, some studies have reviewed the efficacy of simulation training in various surgical fields and found little supporting evidence for improvements in patient-related outcomes [9–11]. In this aspect, a systematic review of VRS would be important to address the effect of simulation training in this field given the steep learning curve of the procedure as well as the strong dependency between operating success and visual outcome. In particular, it would be important to focus on VRS-novices to address skill transfer and examine the potential for implementation of simulation training in a real-life setting.

In order to address if virtual reality simulation can be included in vitreoretinal training of surgical novices, the aim of this study was to perform a systematic review to evaluate the evidence available.

## Methods

This study was conducted and reported in adherence to the Preferred Reporting Items for Systematic Reviews and Meta-Analyses (PRISMA) [17].

In the rest of this study, whenever “student” is mentioned, it refers to a medical student. “Resident” or “fellow” refers to ophthalmological residents or fellows. “Surgeon” refers to a surgeon experienced in VRS.

### Eligibility criteria

The initial inclusion criteria were any study dealing with simulator training of novices in VRS. We then excluded studies which only dealt with laser procedures, did not include VRS novices in their study population, and studies in any other language than English. No specific outcome measures were needed for eligibility, other than some sort of performance measure, being either simulator measured metric or procedural outcomes.

### Literature search

We searched Pubmed, Embase and Cochrane Library using the search string: “(retinal surger* OR vitreoretinal surger*) AND (simulation* OR simulator* OR virtual reality)”. Relevant subject-headings were identified in each database and incorporated in the search. The searches were conducted on March 2nd, 2018 (see Additional file 1 for complete search strategy). Reference lists of studies included after full-text screening was manually searched for additional studies. The Covidence software (Veritas Health Innovation Ltd., Melbourne, Australia) was used for managing references during screening.

### Study selection

Title and abstract screening were done by two authors (R.C.R and A.S.V.). Two authors (R.C.R and A.S.V) then independently full-text reviewed the remaining articles. Eligibility was agreed, and any disagreements were resolved through discussion and mutual consensus.

### Data extraction

A spreadsheet was used to extract the following data items: study aim, study design, number and type of participants, simulator type and model, location and number of institutions, skills trained, control (if present), simulator metrics measured, outcome measures, a summary of results, study conclusion, strengths and limitations, and items needed for quality assessment. Skills trained on the simulator were subcategorized as complex procedures, intraocular navigation or instrument handling. Measured outcomes were subcategorized as skill acquisition, skill assessment, intraocular navigation, performance curves, iatrogenic damage, instrument handling or surveys. We only included skills and outcomes relevant to this review, thus excluding outcomes not related to VRS or simulator performance. The original data extraction was done by one author (R.C.R.) and afterwards reviewed by a second author (A.S.V.). Microsoft Excel 2016 was used for storing and managing extracted data.

### Data simplifications

“Complex procedures” covers any training that resembles real-life surgery, e.g. vitrectomies, membrane peelings or treatment of retinal detachment. “Intraocular navigation” refers to training programs on the EyeSi® Surgical Simulator (VRMagic, Mannheim, Germany), where trainees need to touch orbs varying in size and distance to retina inside the virtual eye. “Skill acquisition” refers to outcomes where training scenarios differ from testing scenarios. “Skill assessment” refers to a simulators cross-sectional ability to assess skills, e.g. differentiating novices from experts. “Performance curves” refers to outcomes measured across multiple sessions on the same device.

### Assessment of quality and risk of Bias

Methodological quality of the included studies was assessed using the Medical Education Research Study Quality Instrument (MERSQI) [18]. This is an assessment tool with ten items divided into six domains: study design, sampling, type of data, validity of evaluation instrument, data analysis, and outcomes. The instrument validation is based on framework proposed by Messick, [19] and includes internal structure, content, and relationship to other variables. MERSQI scoring was done by one author (R.C.R).

The possible risk of bias in the included studies was assessed using the Cochrane Collaboration’s tool for assessing risk of bias, chapter 8.5 in the Cochrane Handbook [20]. The included studies were assessed independently by two authors (R.C.R. and A.S.V.) and judged with low, unclear or high risk of bias for each item. Any disagreements were resolved through mutual consensus.

We have included both randomized and non-randomized studies. The Cochrane bias tool was not developed with non-randomized studies in mind, but the general structure may still be useful when assessing these, in accordance with chapter 13.5.2.3 of the Handbook [20]. When looking at non-randomized studies, selection bias has to be judged differently. We did this by looking at how well the protocol of allocating the participants into their respective groups was described. If the progress was clearly described and predefined, we judged it with a low risk of bias.

## Results

### Study selection

**Fig. 1:**
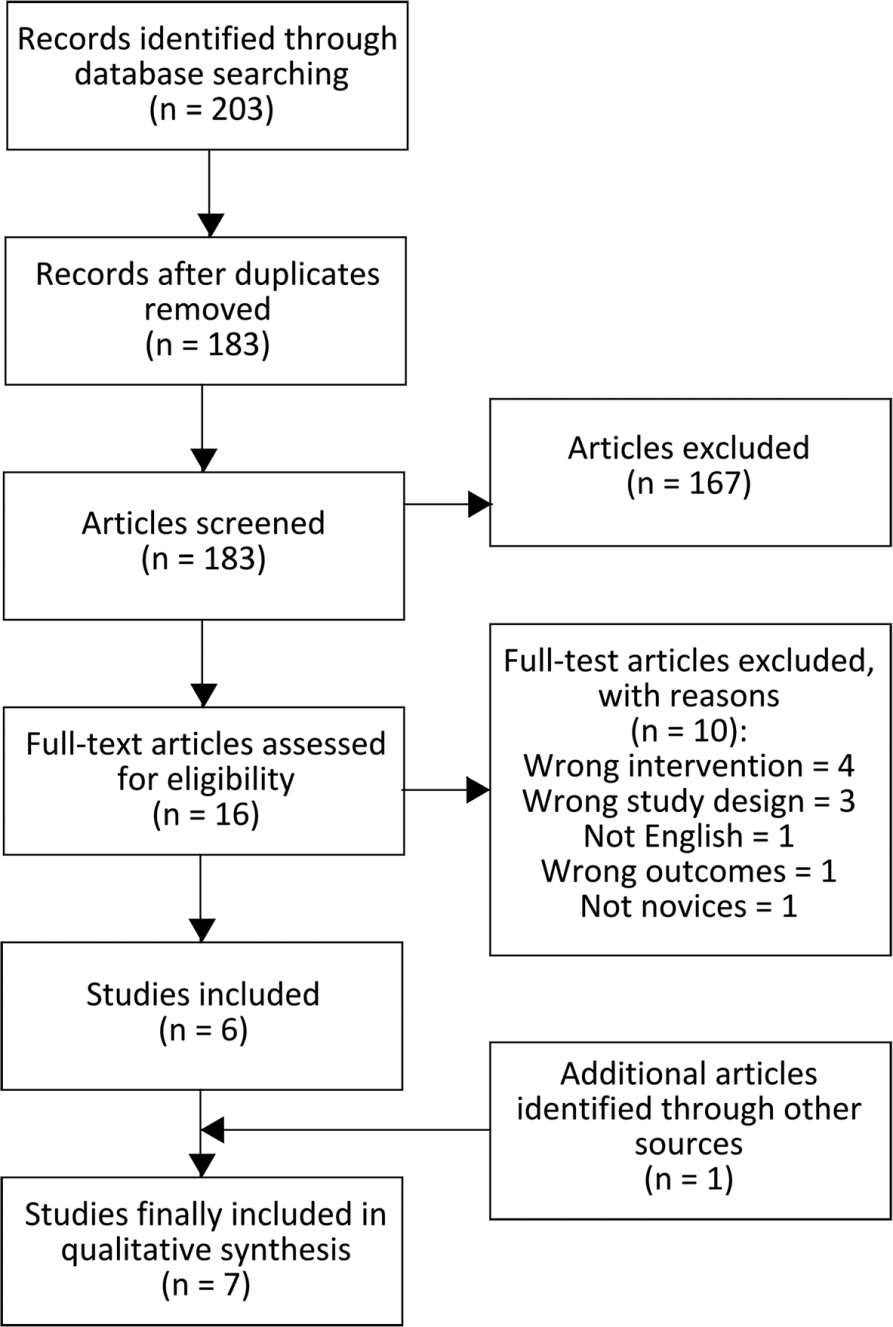
Flowchart of the study selection process

We identified 203 records through the initial searches in Pubmed, Embase and Cochrane Library. After removing duplicates, an additional 167 records were excluded on basis of title and abstract screening, mainly because they were purely descriptive of nature, dealt with non-VRS procedures, or in other languages than English. The exclusion criteria for the remaining 16 articles and a summary of the process are illustrated in Fig. 1. Finally, seven studies were included in this review for qualitative analysis.

### Quality of studies

The highest scoring domains on the MERSQI instrument were type of data and data analysis in which only Yeh et al. [21] did not achieve maximum points, given that their data were non-objective and descriptively analyzed only. Lowest scoring domain was the validity of evaluation instrument. Thomsen et al. [22] was the only to fulfill the criterion for reporting internal structure by testing internal consistency across all modules with Cronbach’s α. Jonas et al., [23] Vergmann et al., [24] and Thomsen et al. [22] adequately reported content and five studies (Rossi et al., [25] Solverson et al., [26] Yeh et al., [21] Vergmann et al., [24] and Thomsen et al. [22]) reported relationships with other variables. Grodin et al. [27] did not score any points in validity of evaluation instrument. (See Additional file 2 for complete MERSQI scoring).

### Risk of Bias

**Fig. 2:**
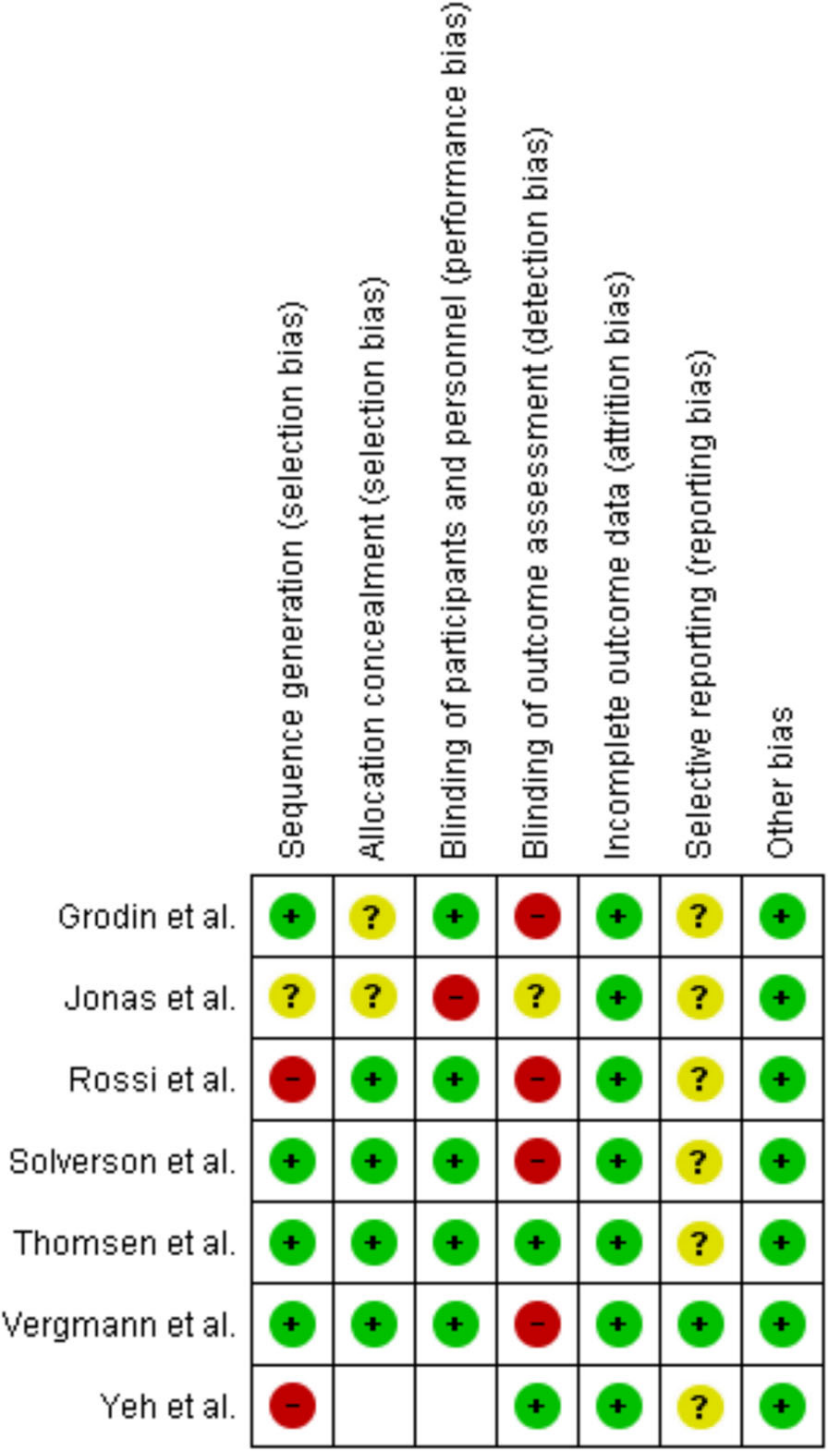
Risk of bias summary. A summary of the authors’ judgements on each risk of bias item for each included study. Green circle = low risk of bias; yellow circle = unclear risk of bias; red circle = high risk of bias; blank = not applicable due to study design. Software used: Review Manager v5.3 (The Nordic Cochrane Centre, The Cochrane Collaboration, Copenhagen, Denmark)

None of the included studies were judged with low risk of bias across all domains. Thomsen et al. [22] was the only study with no items judged with high risk of bias. Blinding of outcome assessment was the worst domain, with four of seven studies judged with high risk of bias. Reporting bias was difficult to judge because of only Vergmann et al. [24] had an available protocol. Yeh et al. [21] had a single-group study design, and because of that, it was not possible to judge performance bias or allocation concealment. A summary of the authors’ judgements of bias in the included studies is shown in Fig. 2.

### Study characteristics

The included studies were published between 2003 and 2017 [21–27]. Rossi et al., [25] Solverson et al., [26] Grodin et al., [27] Vergmann et al. [24] and Thomsen et al. [22] all used the same virtual reality simulator, the EyeSi® Surgical Simulator. Jonas et al. [23] used an unspecified computer-assisted training system. Yeh et al. [21] used a dry-lab simulator, VitRet Eye Model (Philips Studio, Bristol, UK). Table 1 summarizes the study characteristics.

**Table 1:** Characteristics of the included studies

Study characteristics	Studies (Number)	Participants (Number)
All studies	7	191
Study design^a^		
Single group		
Posttest only	1	13
Pre-posttest	1	45
Nonrandomized, multiple groups	4	119
Randomized controlled trials	1	14
Participants		
Medical students	2	26
Medical students / Ophthalmic residents^b^	1	14
Ophthalmic residents	6	101
Ophthalmic fellows	2	15
Vitreoretinal surgeons	5	35
Skills trained^c^		
Vitrectomy	1	13
Membrane peel	4	109
Intraocular navigation	5	133
Instrument handling	3	63
Year of publication		
2003–2008	3	103
2009–2013	2	38
2014–2018	2	50

^a^, as categorized in context with desired outcomes; ^b^, one study reported its participants as medical students or residents without further specification; ^c^, some studies trained multiple skills

### Summary of results

We have conducted a comprehensive, systematic literature search and found seven studies relevant to answer the research question of how simulators can be employed in the training of novices in VRS. We have found that especially the EyeSi® Surgical Simulator can be used to assess and possibly improve basic and complex skills in VRS. Systematic reviews [9, 10, 28] investigating the effect of simulator-based surgical training in other fields than ophthalmology have found similar results.

If we look at our combined results from the included studies, we found evidence that the EyeSi® Surgical Simulator can differentiate novices from experts in VRS. In this differentiation, the most thoroughly tested module was the navigation module, tested by Rossi et al., [25] Solverson et al., [26] Vergmann et al. [24] and Thomsen et al. [22]. Differentiation in the membrane peeling module was tested by Rossi et al., [25] Vergmann et al. [24] and Thomsen et al. [22]. In both modules a statistical significant performance difference was found between novices and experts. All included studies but Yeh et al. [21] reported performance for two or more attempts. Results were that multiple attempts did improve performance and was reported statistical significant by four of the included studies [22–25]. It is difficult to conclude much from the performance curves, as multiple attempts on any device logically should yield improved performance. Solverson et al. [26] and Vergmann et al. [24] reported no improvement in the expert groups, which could be interpreted as the EyeSi® Surgical Simulator closely resembles real surgery. Yeh et al. [21] was the only not using a virtual reality simulator, but instead a dry-lab simulator. Their results were from a questionnaire and correlation between VRS experience and performance measured by a self-developed rating tool. However, it is immensely difficult to say anything in general from these results, as neither dry-lab simulator nor rating tool has been validated before. Table 2 presents a summary of the included studies.

**Table 2:** Summary of skills assessed, measured outcomes and effect

Article	Year	Skills trained	Study type^a^	Participants	Measured outcomes	Summary of effect
Grodin et al. [29]	2008	Complex procedure	Single group pre-post test	29 residents, 10 fellows, 6 surgeons	(1) Performance curves	(1) Improvement (NR)
Jonas et al. [23]	2003	Complex procedure, intraocular navigation	Randomized controlled trial	14 students or residents	(1) Skill acquisition (2) Iatrogenic damage (3) Performance curves	(1) Simulator-trained > no training (NS) (2) Simulator-trained > no training (NS) (3) Improvement*
Rossi et al. [24]	2004	Complex procedure, intraocular navigation	Nonrandomized group comparison	6 students, 24 residents, 14 surgeons	(1) Intraocular navigation (2) Performance curves (3) Skill assessment† (4) Iatrogenic damage	(1) Students < Surgeons* (2) Improvement* (3) Students < Residents*, Residents < Surgeons* (4) Students < Residents < Surgeons (NR)
Solverson et al. [25]	2009	Intraocular navigation	Nonrandomized group comparison	18 residents, 7 surgeons	(1) Intraocular navigation (2) Instrument handling (3) Skill assessment† (4) Performance curves	(1) Residents and fellows < Surgeons* (2) Residents and fellows < Surgeons* (3) Residents and fellows < Surgeons* (4) Residents and fellows improved (NR)
Thomsen et al. [27]	2017	Complex procedure, intraocular navigation, instrument handling	Nonrandomized group comparison	12 residents, 3 surgeons	(1) Skill assessment† (2) Performance curves	(1) Residents < Surgeons* (2) No difference at final, i.e. residents improved*
Vergmann et al. [26]	2017	Complex procedure, intraocular navigation, instrument handling	Nonrandomized group comparison	20 students, 10 residents, 5 surgeons	(1) Instrument handling (2) Intraocular navigation (3) Skill assessment† (4) Performance curves	(1) Students and residents < Surgeons* (2) Students and residents < Surgeons* (3) Students and residents < Surgeons* (4) Students and residents improved*
Yeh et al. [28]	2011	Complex procedure, instrument handling	Single group posttest only	8 residents, 5 fellows	(1) Survey	(1) Three of four statements‡ got positive feedback

NR, significance level not reported; NS, not significant (*p* > 0.05); ^a^, as categorized in context with desired outcomes; *, statistically significant (*p* < 0.05); †, related to multiple outcomes; ‡, “improved understanding of vitreoretinal surgery”, “mimic of basic vitreoretinal surgery”, “helpfulness in vitreoretinal fellowship education”

### Description of individual studies

Due to the heterogeneity of the included studies we give an individual description of each study below.

Grodin et al. [27] investigated whether a training curriculum developed using Systems Approach to Training (SAT) was superior to a traditional curriculum based on standard surgical textbooks in the field. The participants (*n* = 45) were randomized into two groups and both groups performed epiretinal membrane peeling on the EyeSi® Surgical Simulator. Afterwards, they were presented to one of either curriculum, and hereafter performed a second epiretinal membrane peeling. Primary outcome was percentage of epiretinal membrane removed. The SAT instructed group improved from 94 to 97% in epiretinal membrane removed – the traditionally instructed group improved from 86 to 91%. No statistical analysis was done on the performance-improvement of each group from first to second attempt.

Jonas et al. [23] tested how skills transfer from a computer-assisted training system to pars plana vitrectomy in enucleated pig eyes. The participants (*n* = 14) were randomized into two groups that either practiced on the simulator or received no training prior to performing three vitrectomies. Simulator practice included navigation and epimacular membrane peeling. The primary outcome was vitreous removed after 10 min. Results from the three vitrectomies were 45.7% vs. 42.9% (*p* = 0.71), 62.9% vs. 51.4% (*p* = 0.26), and 64.3% vs. 57.1% (*p* = 0.54), simulator-trained group vs. untrained group respectively. Thus, no results showed a statistically significant difference.

Rossi et al. [25] conducted a study investigating correlation between VRS experience and completion time, and performance curves on the EyeSi® Surgical Simulator. The participants were divided into three groups. Group I consisted of students (*n* = 3), residents (*n* = 12) and surgeons (*n* = 7). Group III also included students (n = 3), residents (n = 7) and surgeons (*n* = 6), while Group II only included residents (*n* = 5) and surgeons (n = 1). Group I performed an intraocular navigation task once. Group II repeated the same navigation task ten times. Group III performed a membrane peeling task once. Time to completion was recorded on all tasks. Average completion times in group I was 121.6, 92.5, and 70.6 s for students, residents, and surgeons respectively. The difference between students and surgeons was statistically significant (*p* = 0.004) – difference between students and residents was not (*p* > 0.05). Group II showed a decrease in completion times (*p* = 0.001). In group III students, residents and surgeons had average completion times of 197, 144 and 118.2 s respectively. The differences between students and residents (*p* = 0.05), and residents and surgeons (*p* = 0.003) were statistically significant.

Solverson et al. [26] evaluated the EyeSi® Surgical Simulator as a training and assessment tool. Participants were divided into two groups based on prior VRS experience; novices (*n* = 18) and experts (*n* = 7). Both groups ran three pre-test trials, then repeated five iterations of a navigation task divided into four levels (each level requiring a certain score, before passing to next level). The primary outcome was a “total error” score, calculated for each iteration. “Total error” consisted of scores from “time error”, “odometer error”, and “other error”. Total error showed a difference at baseline between novices and experts with scores of 24.1 and 11.3 respectively (*p* < 0.05). After the final iteration, there was no statistical difference between novices and experts, that had a “Total error score” of 10.2 and 8.4, respectively.

Thomsen et al. [22] investigated whether prior cataract training improves performance in VRS surgery. Twelve residents were randomized to cataract training or no training. An expert group of three surgeons was included. All participants completed eleven vitreoretinal modules (including navigation and epiretinal membrane peeling) on the EyeSi® Surgical Simulator until maximum performance scores were achieved. The cataract trainee group had completed a cataract training program prior to the vitreoretinal modules. A total score from all eleven modules for the first and last repetition was reported, along with procedural time to reach maximum performance. Scores from the first repetition showed that surgeons outperformed cataract trainees (*p* = 0.006) and novices (*p* = 0.003).

Vergmann et al. [24] evaluated the EyeSi® Surgical Simulator as a training and assessment tool. Participants were divided into three groups based on prior VRS experience; students (*n* = 20), residents (*n* = 10) and surgeons (*n* = 5). Each group was instructed in the tasks and completed six vitreoretinal modules (including navigation and internal limiting membrane peeling) twice with a performance evaluation after the first session. The primary outcome was overall score, combining scores from all six modules. The scores were reported for both sessions. In average overall score from first to second session students improved from 134.5 to 272.5 (*p* < 0.01), residents from 254 to 399.5 (*p* = 0.02). Surgeons had no improvement, 405 to 466 (*p* = 1.00). Intergroup comparison of overall score on all three groups showed a statistically significant difference after the second session (*p* < 0.01).

Yeh et al. [21] evaluated a self-developed vitreoretinal training module. Participants (*n* = 13) watched an instructional video twice, then performed VRS tasks on the module, including a core vitrectomy. Participants then answered a post-test questionnaire concerning the training module. Outcome relevant to this review was the answers to the questionnaire. Results were positive feedback on three of four questionnaire statements. The training module helped the participants to better understand basic steps in VRS, mimicked basic VRS, and was deemed helpful in vitreoretinal fellowship education. The module got mixed feedback on whether it mimicked patient tissue.

## Discussion

All included studies but Yeh et al. [21] measured positive performance curves. Rossi et al., [25] Solverson et al., [26] Thomsen et al. [22] and Vergmann et al. [24] reported that the EyeSi® Surgical Simulator was able to differentiate students from residents and residents from surgeons. Jonas et al. [23] showed that training on their simulator was superior to no training.

The MERSQI tool was included to evaluate methodological quality, but Cook and Reed [29] advises that one should take caution looking at scores, and instead focus on item-specific codes, preferably individual instrument scores. When assessing individual item scores, validity of the evaluated instruments in the included studies was low. Thomsen et al. [22] was the only study to adequately report data for all three items. Vergmann at el. [24] reported adequately for two of three items – content and relationships to other variables. Jonas et al. [23] reported content through a detailed description of the simulated training scenarios. Yeh et al., [21] Solverson et al., [26] and Rossi et al. [25] reported relationships to other variables by how well the simulator differentiated the participants based on VRS experience. We speculate that researchers have become aware of the need to investigate and report instrument validity, since the studies by Thomsen et al. [22] and Vergmann et al. [24] are the most recent published. Hence, the reason the remaining studies are lacking instrument validity might simply be ignorance to the knowledge on the subject. A general lack of instrument validity in studies assessing medical education is previously reported by Cook et al. [30]. Validity is essential to assessments in medical education, [29] which otherwise has very low intrinsic meaning. [31]

An article by Gallagher et al. [32] defines six different types of validation benchmarks:

1. Face validity: does the instrument in question seem appropriate? A subjective expert validation.
2. Content validity: a more detailed expert review of the different instrument items. Does the instrument resemble what it strives to? Still a subjective validation.
3. Construct validity: an evaluation of whether an instrument measures what it was designed to. Usually tested as the ability to differentiate novices from experts.
4. Concurrent validity: do the test scores from the instrument in question relate to those of a different instrument, purporting to measure the same construct?
5. Discriminate validity: a more sophisticated differentiation than that of construct validity. An evaluation of how test scores and specific factors correlate.
6. Predictive validity: an evaluation of the instruments ability to predict actual performance.

Proving validity is a vital part of assessing simulator training, [32] and using the definitions given by Gallagher et al., [32] the majority of the included studies provide construct validity – Rossi et al., [25] Vergmann et al., [24] Solverson et al., [26] and Thomsen et al. [22] It could be argued that Jonas et al. [23] provides concurrent validity, but one should take caution drawing conclusions about real operating room performance from performance on enucleated pig eyes. [4] Yeh et al. [21] provides face validity, which is usually used in the early stages of developing a new training instrument. [32] Unfortunately, face validity is very difficult to utilize when trying to draw broader conclusions, as it is highly subjective. The study by Grodin et al. [27] was difficult to categorize in terms of validity. The instrument in question is actually their training curriculum [27] and therefore does not contribute to any simulator validation. None of the included studies provides discriminate or predictive validity [32]. Cook [33] questions the relevance of construct validity, as confounding is inevitable and such studies should be interpreted with caution. At the same time, construct validity is a necessity, but it is when such analysis fail that it is interesting [33]. As none of the included studies fail to prove construct validity, it is imperative to focus on higher levels of validity. An example of predictive validity was given by a recent study by Deuchler et al., [34] which investigated the performance impact of simulator warmup prior to real surgery among VRS surgeons. Unfortunately, transferring their design to studies involving novices could be difficult due to potential ethical issues. However, studies investigating concurrent validity should emerge in the coming years.

The risk of bias assessment showed that none of the included studies fulfilled all the criteria set by the Cochrane Collaboration to receive a judgement of low risk of bias across all domains. This may have multiple reasons. Only one of the included studies provided a protocol [24], making it not possible to judge the rest of the included studies regarding reporting bias. Five of seven items judged with high risk of bias were blinding of either participants and personnel or outcome assessment. In all cases this was because of inadequate reporting of any blinding at all, displaying ignorance or lacking awareness of the importance of the subject. Generally, the risk of bias was low across all included studies, but the lack of blinding is alarming, and because of that the results should be interpreted with caution.

It is interesting to look at the design of training versus no training, as Jonas et al. [23] utilizes. It would be surprising not to find the trained group superior, as any training seems better than none [11]. A systematic review by Zendejas et al. [28] investigated the evidence behind laparoscopic surgical training. They found similar results comparing simulation and no training, but when comparing virtual reality and box trainers, there were no clear favors. This indicates the importance of studies comparing different training methods or simulators. We do acknowledge that testing versus a completely untrained control is one of the first steps in the validation of a new training method. The study by Jonas et al. [23] indicate that the EyeSi® Surgical Simulator is a viable teaching tool in VRS. The newer studies [22, 24–26] in this review has continued to enhance validity to the simulator. In line with our findings, a recent systematic review of orthopedic simulators by Morgan et al., [3] indicates a need to enhance validation. We emphasize that future studies should focus on skill transfer, possibly from simulator to an operating room-like setting.

Regarding specific outcomes, the included studies had some degree of heterogeneity. Jonas et al. [23] reported performance in vitrectomies on enucleated pig eyes. Four studies [22, 24–26] reported how well participants of different VRS experience differentiated in simulator-measured metrics, reported as “error scores”, [26] total scores across eleven [22] or six modules, [24] or as time to completion [25]. Most studies measured performance curves across two or more simulator sessions but again, there was variance to how it was reported, and some studies did not provide numerical values. Thus, the heterogeneity excludes quantitative analyses across the studies. This is a general problem in reviews regarding simulation, making it difficult to draw strong conclusions [3, 35–37]. It should be noted that Grodin et al., [27] Yeh et al., [21] and Thomsen et al. [22] had other primary study purposes, than directly investigating simulated VRS training among novices. They were still included because they matched the inclusion criteria.

Even though we only found seven relevant studies in the field of VRS, studies investigating simulation in the anterior segment of the eye are numerous [11]. Sikder et al. [12] has made a review on simulation training in cataract surgery. The results regarding construct and concurrent validity are similar to those of our own. A systematic review by Thomsen et al. [11] on simulation-based training in ophthalmology showed a general lack of instrument validity and patient-related outcomes. Another study by Thomsen et al. [38] investigated correlation between past real cataract surgery and present performance on EyeSi® Surgical. The results showed a strong correlation between past and present performance. This further supports EyeSi® Surgical as a useful tool in the field of cataract surgery, which hopefully can be transferred to VRS.

### Strengths and limitations

This study has certain strengths. First, we are the first to conduct a systematic review concentrated on simulator training among novices in VRS. Second, the study was conducted in accordance with PRISMA [17].

We might have some limitations. Only including English literature might bias our results since some studies in German were excluded. We did not search for any gray, unpublished literature, which might explain why we mainly found positive results. The number of relevant studies was small and only included one study randomized regarding VRS novices. MERSQI scoring might be insecure, because of limited available guidelines. Lastly, the results from our risk of bias assessment show a possible risk of bias among the included studies, which weakens our conclusion.

### Perspectives

Results from the included studies indicate that simulators give an opportunity to acquire new surgical skills. Although future studies need to confirm how it translates to a clinical setting. The findings from Solverson et al. [26] and Vergmann et al. [24] showing that surgeons did not have performance improvement may suggest that the EyeSi® Surgical Simulator resembles something very close to reality. On the other hand, it could also indicate that the tested modules were too easy for the surgeons.

In a clinical setting, the results from this review suggest, that simulators could be used to assess VRS skills. This could be used in the education of new surgeons. An objective measurement of skills would allow a more individualized education program, targeting specific deficits. Objective simulator metrics could decide whether a trainee has acquired sufficient surgical skills, before operating on real patients.

In future studies investigating simulation training in VRS, we hypothesize that it would be beneficial to focus on randomized controlled trials. Randomized controlled trials are expensive and require a lot of resources but are needed to rule out bias. An acknowledgement from researchers that validity is imperative to education research is also needed. Frameworks as those proposed by Messick [19] must be considered when designing and reporting future studies [11]. Risk of bias across the included studies are high or unclear, and future studies should make sure to adequately report methods to avoid bias, including publishing their protocols.

Selwyn et al. [39] has found that surgical errors have multiple reasons other than inexperience or lack of technical competence. It could be interesting to see future research focus on non-technical skills, like communication, leadership or handling of stress and crisis [6, 9].

## Conclusion

This systematic review concludes that simulators in VRS currently are useful as assessment-tools and may be able to teach the complex techniques required in VRS. We were unable to rule out a significant risk of bias which might influence our conclusion. The studies investigating simulation training among novices in VRS have almost unanimously focused on the EyeSi® Surgical Simulator. These studies suggest that the simulator can assess an array of basic and complex VRS skills. Currently, no evidence supports that simulator-based training of novices in VRS is capable of transferring to the operating room. Supporting evidence is lacking in terms of instrument validity. This systematic review proposes that future studies continue to enhance instrument validity. An effort should be put into streamlining study designs and validation terms. The streamlining will make it possible to compare studies and draw stronger conclusions. The next step is to establish concurrent validity, proving transfer from a simulator to another validated testing scenario. When concurrent validity is supported by strong evidence, future research could focus on investigating simulator to operating room skill transfer.

## Additional files

Additional file 1: “Search strategies” and include detailed information on how the literature search was conducted in this study. (PDF 106 kb)

Additional file 2: “MERSQI scores” and includes a table with the individual MERSQI scores on all included studies. (PDF 171 kb)

## Abbreviations

MERSQI: Medical Education Research Study Quality Instrument
PRISMA: Preferred Reporting Items for Systematic Reviews and Meta-Analyses
VRS: Vitreoretinal surgery
