# Supplementary material for: Simulation training in vitreoretinal surgery: a systematic review
Source: BMC Ophthalmol. 2019 Apr 11;19:90. doi: 10.1186/s12886-019-1098-x (PMC6460826; doi:10.1186/s12886-019-1098-x)
Supplement: Supplementary file 1 — “Search strategies” and include detailed information on how the literature search was conducted in this study. (PDF 106 kb) [file 12886_2019_1098_MOESM1_ESM.pdf]

# Search Strategies

Search strategy: PubMed (02-03-2018)

| | | |
|---|---|---|
| 01. | Vitreoretinal surgery [MeSH Terms] | 373 results |
| 02. | Simulation training [MeSH Terms] | 5974 results |
| 03. | High fidelity training [MeSH Terms] | 119 results |
| 04. | Vitreoretinal surgery | 3401 results |
| 05. | Retinal surgery | 41979 results |
| 06. | Simulation | 322803 results |
| 07. | Simulator | 13983 results |
| 08. | Virtual reality | 7973 results |
| 09. | 4 OR 5 | 42597 results |
| 10. | 6 OR 7 OR 8 | 334648 results |
| 11. | 10 AND 9 | 150 results |
| 12. | Virtual reality [MeSH Terms] | 160 results |
| 13. | 2 OR 3 OR 11 | 6121 results |
| 14. | 12 AND 1 | 1 results |
| 15. | 11 OR 14 | 150 results |

Final search: (Vitreoretinal surgery [MeSH Terms] AND (Simulation training [MeSH Terms] OR High fidelity training [MeSH Terms] OR Virtual reality [MeSH Terms])) OR ((Vitreoretinal surgery OR Retinal surgery) AND (Simulation OR Simulator OR Virtual reality))

Search strategy: EMBASE (Ovid) (02-03-2018)

| | | |
|---|---|---|
| 01. | Exp simulation training/ | 2129 results |
| 02. | Exp simulation/ | 237539 results |
| 03. | Vitreoretinal surgery/ | 863 results |
| 04. | Retinal surger*.mp. | 657 results |
| 05. | Vitreoretinal surger*.mp. | 2348 results |
| 06. | Simulation*.mp. | 368090 results |
| 07. | Simulator*.mp. | 22316 results |
| 08. | Virtual reality/ | 12544 results |
| 09. | Virtual reality.mp. | 16073 results |
| 10. | 1 OR 2 OR 8 | 249411 results |
| 11. | 3 AND 10 | 17 results |
| 12. | 4 OR 5 | 2914 results |
| 13. | 6 OR 7 OR 9 | 389880 results |
| 14. | 12 AND 13 | 31 results |
| 15. | 11 OR 14 | 31 results |

Final search: ((Exp simulation training/ OR Exp simulation/ OR Virtual reality/) AND Vitreoretinal surgery/) OR ((Retinal surger*.mp. OR Vitreoretinal surger*.mp.) AND (Simulation*.mp. OR Simulator*.mp. OR Virtual reality.mp.))

Search strategy: Cochrane Library (02-03-2018)

| | | |
|---|---|---|
| 01. | [mh Vitreoretinal Surgery] | 28 results |
| 02. | [mh Simulation Training] | 634 results |
| 03. | [mh Virtual Reality] | 11 results |
| 04. | Retinal surgery | 1676 results |
| 05. | Vitreoretinal surgery | 265 results |
| 06. | Simulation | 8841 results |
| 07. | Simulator | 1699 results |
| 08. | Virtual reality | 1591 results |
| 09. | 2 OR 3 | 642 results |
| 10. | 1 AND 9 | 0 results |
| 11. | 4 OR 5 | 1789 results |
| 12. | 6 OR 7 OR 8 | 10625 results |
| 13. | 11 AND 12 | 22 results |
| 14. | 10 and 13 | 22 results |

Final search: ([mh "Vitreoretinal Surgery"] AND ([mh "Simulation Training"] OR [mh "Virtual Reality"])) OR ((Retinal surgery OR Vitreoretinal surgery) AND (Simulation OR Simulator OR Virtual reality))
