# Supplementary material for: Simulation training in vitreoretinal surgery: a systematic review
Source: BMC Ophthalmol. 2019 Apr 11;19:90. doi: 10.1186/s12886-019-1098-x (PMC6460826; doi:10.1186/s12886-019-1098-x)
Supplement: Supplementary file 2 — “MERSQI scores” and includes a table with the individual MERSQI scores on all included studies. (PDF 171 kb) [file 12886_2019_1098_MOESM2_ESM.pdf]

MERSQI Scores

Designated scores of individual MERSQI items.

Individual MERSQI domains, items and requirements.	*Item score**	Yeh et al.	Jonas et al.	Thomsen et al.	Grodin et al.	Vergmann et al.	Rossi et al.	Solverson et al.
Study design[a]								
Singlegroup posttest only	*1*	1						
Singlegroup pre- and posttest	*1.5*				1.5			
Nonrandomized, multiple groups	*2*			2		2	2	2
Randomized controlled trial	*3*		3					
Sampling								
Number of institutions studied								
1	*0.5*	0.5	0.5	0.5		0.5	0.5	0.5
2	*1*							
>2	*1.5*				1.5			
Response rate, %								
<50 or not reported	*0.5*							
50-74	*1*	1						
>74	*1.5*		1.5	1.5	1.5	1.5	1.5	1.5
Type of data								
Assessment by participant	*1*	1						
Objective measurement	*3*		3	3	3	3	3	3
Validity of evaluation instrument								
Internal structure								
Not reported	*0*	0	0		0	0	0	0
Reported	*1*			1				
Content								
Not reported	*0*	0			0		0	0
Reported	*1*		1	1		1		
Relationships to other variables								
Not reported	*0*		0		0			
Reported	*1*	1		1		1	1	1
Data analysis								
Appropriateness								
Inappropriate for design or type of data	*0*							
Appropriate for design or type of data	*1*	1	1	1	1	1	1	1
Complexity of analysis								
Descriptive only	*1*	1			1			
Beyond descriptive	*2*		2	2		2	2	2
Outcomes								
Satisfaction, attitudes, perceptions, opinions	*1*	1						
Knowledge, skills	*1.5*		1.5	1.5	1.5	1.5	1.5	1.5
Behaviors	*2*							
Patient/health care outcome	*3*							

The highest possible score was 18. The six MERSQI domains and total score are highlighted. MERSQI; Medical Education Research Study Quality Instrument; [a], as categorized in context with desired outcomes; *, the score that would be assigned to a study given it fulfilled the item requirements.
